# Assessing SARS-CoV-2 Testing Adherence in a University Town: Recurrent Event Modeling Analysis

**DOI:** 10.2196/48784

**Published:** 2024-04-17

**Authors:** Yury E García, Alec J Schmidt, Leslie Solis, María L Daza-Torres, J Cricelio Montesinos-López, Brad H Pollock, Miriam Nuño

**Affiliations:** 1 Department of Public Health Sciences University of California Davis, CA United States; 2 Clinical and Translational Science Center University of California Davis, CA United States

**Keywords:** Healthy Davis Together, COVID-19, COVID-19 surveillance program, community surveillance, HDT: HYT, Healthy Yolo Together, SARS-CoV-2, severe acute respiratory syndrome coronavirus 2, coronavirus, demographic, demographics, testing, adherence, compliance, USA, United States, response program, response programs, engagement, participation, infectious, trend, trends, community based, surveillance, public health, infection control, PCR, polymerase chain reaction, RT-qPCR, reverse transcription quantitative polymerase chain reaction, viral, virus, viruses

## Abstract

**Background:**

Healthy Davis Together was a program launched in September 2020 in the city of Davis, California, to mitigate the spread of COVID-19 and facilitate the return to normalcy. The program involved multiple interventions, including free saliva-based asymptomatic testing, targeted communication campaigns, education efforts, and distribution of personal protective equipment, community partnerships, and investments in the local economy.

**Objective:**

This study identified demographic characteristics of individuals that underwent testing and assessed adherence to testing over time in a community pandemic-response program launched in a college town in California, United States.

**Methods:**

This study outlines overall testing engagement, identifies demographic characteristics of participants, and evaluates testing participation changes over 4 periods of the COVID-19 pandemic, distinguished by the dominant variants Delta and Omicron. Additionally, a recurrent model is employed to explore testing patterns based on the participants’ frequency, timing, and demographic characteristics.

**Results:**

A total of 770,165 tests were performed between November 18, 2020, and June 30, 2022, among 89,924 (41.1% of total population) residents of Yolo County, with significant participation from racially or ethnically diverse participants and across age groups. Most positive cases (6351 of total) and highest daily participation (895 per 100,000 population) were during the Omicron period. There were some gender and age-related differences in the pattern of recurrent COVID-19 testing. Men were slightly less likely (hazard ratio [HR] 0.969, 95% CI 0.943-0.996) to be retested and more likely (HR 1.104, 95% CI 1.075-1.134) to stop testing altogether than women. People aged between 20 and 34 years were less likely to be retested (HR 0.861, 95% CI 0.828-0.895) and more likely to stop testing altogether (HR 2.617, 95% CI 2.538-2.699). However, older age groups were less likely to stop testing, especially those aged between 65-74 years and 75-84 years, than those aged between 0 and 19 years. The likelihood of stopping testing was lower (HR 0.93, 95% CI 0.889-0.976) for the Asian group and higher for the Hispanic or Latino (HR 1.185, 95% CI 1.148-1.223) and Black or African American (HR 1.198, 95% CI 1.054-1.350) groups than the White group.

**Conclusions:**

The unique features of a pandemic response program that supported community-wide access to free asymptomatic testing provide a unique opportunity to evaluate adherence to testing recommendations and testing trends over time. Identification of individual and group-level factors associated with testing behaviors can provide insights for identifying potential areas of improvement in future testing initiatives.

## Introduction

The COVID-19 pandemic presented major challenges and demanded effective public health responses involving sustained behavior change, stay-at-home restrictions, face coverings, testing, and vaccination. Public health guidelines are a critical first line of a pandemic response but rely on compliance with evolving recommendations and restrictions. Trust and compliance are driven by complex factors, including individuals’ beliefs [1], risk perception [2,3], trust in government [4], and demographic factors [5-7] that may vary throughout a pandemic, making containment particularly challenging.

Widespread testing and timely diagnosis are critical for pandemic control and preparedness. The deployment of clinical testing on a massive scale (“mass testing” [8]) was one of the essential control measures for curtailing the burden of the COVID-19 pandemic, particularly during its early phases [9]. Early detection of cases can limit viral transmission by isolation, quarantine, and contact tracing [10-12]. New preventive and surveillance mechanisms surged throughout the pandemic, including vaccine programs, wastewater surveillance [13], and at-home COVID-19 tests [14]. Both the virus itself and protective behaviors changed throughout the waves of the pandemic [1], complicating long-term interventions. Government and public health officials implemented health contingencies that were adaptable and flexible to reduce socioeconomic and long-term health burden fatigue [15,16].

Understanding how peoples’ adherence to preventive measures changes over time can guide policy makers as they amend strategies to revitalize public health strategies in future outbreaks. Studies of previous natural disasters suggest that people’s perceptions of risk and their responses to such risk may vary between individuals. Gender, age, socioeconomic status, personal experience of a natural hazard, and trust in authorities affect people’s responses to catastrophic events [6,17,18]. For the COVID-19 pandemic, a growing body of research reported factors associated with adherence and nonadherence to COVID-19 preventive measures [1,19-21]. Specifically, age [5], gender [6,7], higher education level [22], marital status, and having children [23] are all associated with adherence measures.

Healthy Davis Together (HDT) [24] was a program implemented in the city of Davis, California, as an effort to mitigate the spread of COVID-19 and facilitate the return to normalcy. Launched in September 2020, HDT focused on curtailing the pandemic burden among Davis residents and workers. In July of 2021, the program was expanded to support residents of the wider Yolo County in a rebranded effort, Healthy Yolo Together (HYT). It employed a multipronged approach, including engaging the community, implementing effective testing and contact tracing measures, promoting health education, and fostering collaboration with stakeholders.

HDT implemented comprehensive interventions that combined disease control measures and the promotion of health-conscious behaviors. Polymerase chain reaction (PCR) testing accessibility was enhanced with the establishment of new testing locations offering voluntary and complimentary SARS-CoV-2 testing. HDT engaged over 200 public health ambassador students dedicated to advocating for healthy behaviors. Mass communication campaigns were promoted across diverse media platforms to encourage testing and the adoption of health-promoting behavior. The program provided incentives to promote health-promoting behaviors. Collaborations were established with local businesses to implement and adjust safety protocols. The program fostered stronger partnerships among the university, city, county, and private and community organizations. In addition, HDT implemented citywide subsewer shed–level wastewater monitoring for early detection. The program maintained an analytical team tasked with real-time data analysis to pinpoint communities or specific locations requiring increased testing implementation. This analytical approach complemented the program’s health behavior strategies.

In this study, we thoroughly examine testing behavior within the HDT program, emphasizing patterns of testing throughout. We use regression modeling for recurrent events to characterize testing behavior within the HDT program. This method enables us to examine individual testing events and current testing over time, providing insights about the participants’ testing patterns and testing adherence throughout the program’s duration.

## Methods

### Data Description

**Figure 1:**
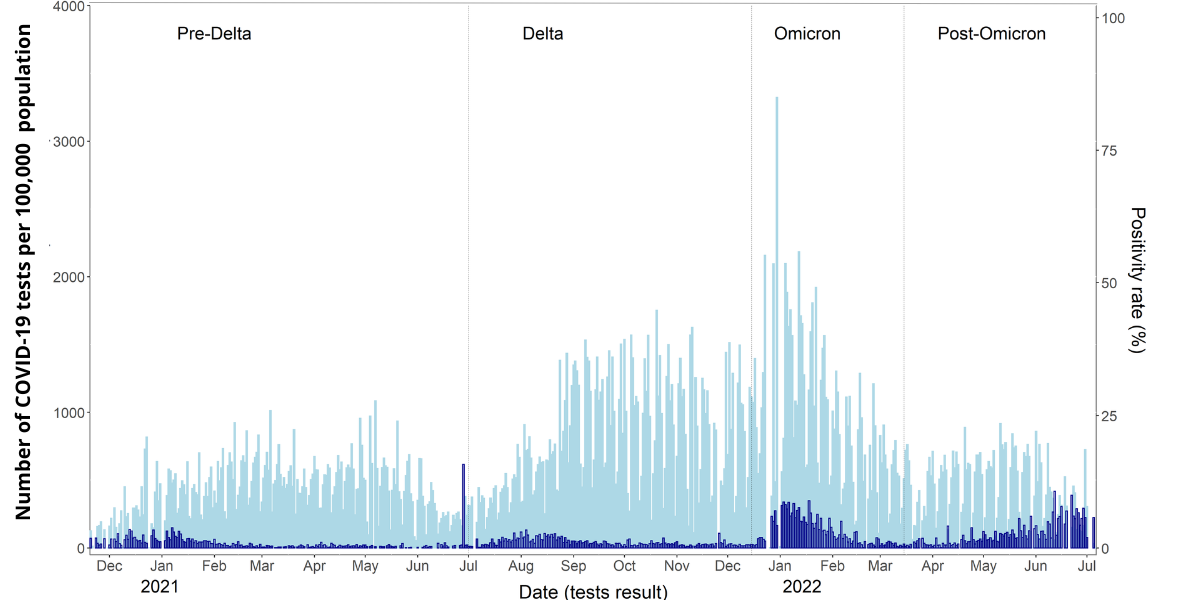
Tests performed per 100,000 population (light blue) and positive test rates (dark blue) daily from November 18, 2020, to June 30, 2022. Free asymptomatic testing was accessible to Yolo County residents in July 2021. Vertical lines mark 4 distinct periods characterized by dominant SARS-CoV-2 variants.

Testing participation was aggregated and subdivided into distinct periods according to the dominant variants Delta and Omicron: pre-Delta (November 18, 2020, to June 13, 2021), Delta (June 14, 2021, to December 20, 2021), Omicron (December 21, 2021, to March 15, 2022), and post-Omicron (March 16, 2022, to June 30, 2022). These periods were based on the most common variant reported by the California Department of Public Health [25] (Figure 1). Hereafter, the Delta B.1.617.2, Omicron B.1.1.529, and subsequent Omicron BA.2, BA.3, BA.4.266, and BA.5 variants will be referred to as Delta, Omicron, and post-Omicron, respectively. During the pre-Delta period, HDT primarily served the city of Davis. Subsequently, at the onset of the Delta variant in July 2021, testing access was extended to encompass Yolo County as a whole.

Yolo County has an estimated population of 218,774 individuals, with 106,002 males and 112,772 females. Among them, approximately 68,640 reside in the city of Davis. Throughout the program, 770,165 tests were administered to 89,924 unique individuals, including 53,869 residents of the city of Davis. A significant number of participants in Davis were linked to the University of California, Davis. The university enforced weekly testing mandates starting in the fall of 2020 for those who were physically present on campus.

HDT program participants were given a unique ID, which allowed us to distinguish between new and returning individuals. An individual can be classified as a unique participant for a specific period (eg, Delta) if they underwent testing at least once during that period, regardless of whether they were also tested during another period. However, for the overall program analysis, each participant was counted only once, regardless of how many times they were tested across multiple periods. New participants are individuals who underwent testing for the first time. Total tests refer to the overall number of tests conducted within a specific period or in total. Positive tests denote the total count of tests yielding a positive result, distinct from participants with a positive result, which indicates the number of individuals who had at least one positive test during a specified period.

### Ethical Considerations

This study used deidentified data and received an exemption from institutional review by the University of California Davis Office of Research.

### Diagnostic Tests

Saliva tests were conducted using an innovative high-throughput quantitative reverse transcriptase–PCR method [24]. Most tests (98.4%) comprised of saliva tests while the remaining 1.6% consisted of BinaxNOW (Abbott) rapid antigen tests administered to symptomatic individuals. Employing saliva samples for quantitative reverse transcription PCR tests enhances the feasibility of widespread asymptomatic testing in the community, as it incurs lower monetary and labor costs with only a slight decrease in sensitivity [26].

### Statistical Models

We used regression modeling for recurrent events to analyze testing behavior, given that participants underwent multiple tests at irregular intervals. This modeling framework facilitates the analysis and interpretation of testing recurrence, providing insights into factors influencing testing adherence, frequency, and consistency among participants. Additionally, trends, fluctuations, and potential predictors of testing behavior can be identified, offering insights for optimizing testing strategies, resource allocation, and public health interventions to promote regular and sustained testing participation. The *reReg* library in R (R Foundation for Statistical Computing) was used to analyze the temporal dynamics of testing behavior [27,28].

The regression Cox model for recurrent events extends the traditional Cox proportional hazards model, which is commonly used for analyzing time-to-event data in survival analysis, to handle recurrent event data. Additionally, this modeling framework enables the examination of covariate effects on the rate of event occurrence while properly addressing the correlation among recurrent events within the same individual. This model, also known as the Andersen-Gill model, can be represented as follows:

*λ_i_* (*t*) = *λ*_0_ (*t*) × exp (*β*_1_*X_i_*_1_ + *β*_2_*X_i_*_2_ +...+ *β_p_X_ip_*) **(1)**

where *λ_i_* (*t*) represents the hazard rate for individual *i* at time *t*. *λ*_0_ (*t*) is the baseline hazard rate at time *t*. *β*_1_*X_i_*_1_ + *β*_2_*X_i_*_2_ +...+ *β_p_X_ip_* denotes the hazard ratio (HR) associated with the covariates *X_i_*_1,_ *X_i_*_2_,..., *X_ip_* with *β*_1,_ *β*_2_,…, *β_p_* representing the regression coefficients for the covariates included in the model. This formula expresses the hazard rate for everyone as a function of the baseline hazard rate and the covariates, with the regression coefficients determining the impact of the covariates on the hazard rate. We assumed noninformative censoring, suggesting that the reasons people decided to take part in testing during the program are not related to why they might eventually stop testing altogether.

### Mean Cumulative Function

The Mean Cumulative Function (MCF) denotes the expected cumulative count of tests up to a specific time point, considering both the occurrence and timing of test events. The MCF was calculated by age and racial or ethnic groups. We also apply the terminal event process Cox model to examine the occurrence of complete cessation of testing while accounting for covariates.

### Data Preprocessing

For this study, individuals who undergo COVID-19 testing at least once a week are selected. Each selected individual is treated as a single event, even if they receive multiple tests within the same week. This study includes 61,363 participants who have undergone testing on at least 2 occasions (2 different weeks). The event for each participant begins with their first test and ends during the subsequent week of another test. A test occurrence is marked as 1, while the status remains 0 to signify ongoing participation. The final testing instance for each participant is identified, and the event of the subsequent week is marked as 0 to indicate a censoring event.

## Results

### Description of Testing Trends

HDT conducted 770,165 tests among 89,924 residents of Yolo County (approximately 41.1% of the Yolo population, 218.793), of which 53,869 individuals reported a zip code in the city of Davis (approximately 78.4% of Davis population, 68.710). Testing coverage was most significant in the city of Davis, where the program originated, and efforts were first focused. Figure 1 illustrates the total daily tests per 100,000 population and positive test rates observed in Yolo County throughout this study. On June 28, 2021, the positivity rate surged, likely because there were fewer tests reported on that day.

Overall, 12,626 tests were positive among 11,545 unique individuals, for an aggregated test positivity rate of 1.64% and a case rate of 13%. The average daily number of tests conducted per 100,000 population was 422 (SD 210) during the pre-Delta period, 746 (SD 449) during Delta, 895 (SD 518) during Omicron, and 435 (SD 254) during the post-Omicron period. A summary of tests and positive cases per study period is reported in Table 1.

**Table 1:** Summary of testing participation for each period of this study and overall.

Characteristics		Pre-Delta	Delta	Omicron	Post-Omicron	Overall
**Tests**						
	Duration of the testing period (days)	208	190	85	107	590
	Total tests, n (%)	191,904 (24.9)	309,928 (40.2)	166,482 (21.6)	101,851 (13.2)	770,165 (100)
	Average tests per day (SD)	922.6 (460)	1631.2 (983)	1958.6 (1333)	951.9 (556)	1305.4 (919)
	Average tests per 100,000 mean population per day (SD)	421.7 (210)	745.6 (449)	895.3 (518)	435.1 (254)	596.7 (420)
**Participants**						
	Overall participants	39,819	58,920	46,545	23,566	89,924
	New participants, n (%)	39,819 (100)	33,814 (57.4)	13,503 (29)	2788 (11.8)	89,924
**Cases**						
	Total positive tests	1197	2817	6351	2261	12,626
	Test positivity rate (%)	0.62	0.91	3.81	2.22	1.64
	Percentage of all positives, %	9.5	22.3	50.3	17.9	100
	Positives among participants, n	1138	2577	5862	2142	11,719
	Positives among new participants, n (%)	1138 (100)	2570 (99.7)	5757 (98.2)	2080 (97.1)	11,545 (98.5)

Total tests conducted, participation, recruitment, and positive test tallies across pandemic periods are displayed in Table 1. For instance, during pre-Delta, 191,904 tests were conducted, which corresponds to 24.9% (n=770,165) of the total tests conducted in the entire program. During Delta, 2570 positive cases were detected in new participants, corresponding to 22.3% (n=11,545) of the total of unique people who resulted as positive.

Changes in policy at the state and local level, social expectations and behavior, the emergence of new strains, and differences in the intensity of testing lead to differing participation rates across pandemic periods. In total, HDT or HYT served 89,924 unique individuals across the entire program. A total of 39,819 (44.3%) participated in testing during pre-Delta, 58,920 (65.5%) during Delta, 46,545 (51.8%) during Omicron, and 23,566 (26.2%) during the post-Omicron period. Further, 61,363 individuals, equivalent to 68.2% of all participants, took part in the testing process multiple times. A longer period of days did not mean a higher number of participants. The highest participation of people was during the Delta period.

Omicron had the highest number of positive cases despite being the shortest period in the program. Out of all positive cases, 6351 (50.3%) were observed during Omicron. On average, 1958 (SD 1333; ∼895, SD 518, tests per 100,000) daily tests were performed at a test positivity rate of 3.81% (Table 1). The enrollment of new participants decreased with each subsequent period: 39,819 individuals were engaged in the first stage of the program (pre-Delta), 33,814 during Delta, 13,503 during Omicron, and 2788 during post-Omicron. Most positive tests (a total of 2080, 97.1%) were from new participants (Table 1).

**Figure 2:**
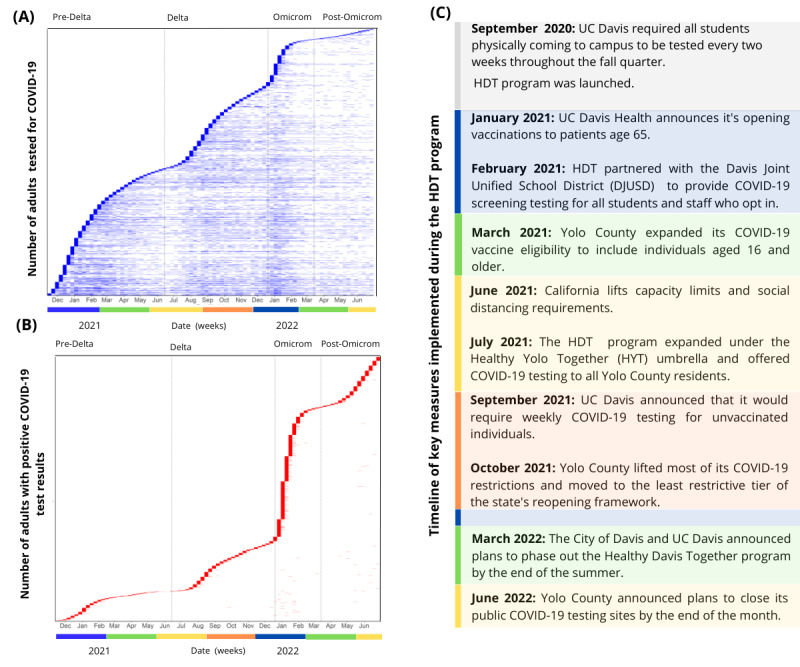
Testing uptake (A) and positive test results (B) among Yolo County residents aged 18 and older (N=67,083) during this study period. Panel C outlines the key measures enacted during the Healthy Davis Program, depicted with color bars representing the seasons: winter (blue), spring (green), summer (yellow), and fall (orange). Vertical lines delineate 4 periods of dominant SARS-CoV-2 variants. DJUSD: Davis Joint Unified School District; HDT: Healthy Davis Together; HYT: Healthy Yolo Together; UC: University of California.

HDT or HYT collaborated with multiple school districts in Yolo County to provide free weekly testing to students. Excluding participants under 19, there were 67,083 participants from the community; among them, 8774 (13%) individuals tested positive, and 422 (4.8%) had positive results in two or more weeks. Further, 265,000 (34.4%) underwent testing once, while participation ranged from 1 to 83 weeks, with a median of 7.5 (IQR 7) weeks. Figure 2A illustrates uptake in testing by individuals, showing the weeks they underwent testing and information on when everyone was initially tested. It is noticeable that at the beginning of each testing wave, there is typically a higher flux of new participants signing up each week. It also enables the observation of participants’ ongoing engagement throughout the program. Figure 2B illustrates the weeks in which an individual received at least one positive test result, showing a notable increase in positive cases during the Omicron wave. Figure 2C describes key measures implemented during the Healthy Davis Program, with color bars representing the seasons: winter (blue), spring (green), summer (yellow), and fall (orange).

Testing rates between male and female residents varied across 4 distinct periods and overall. Female participants underwent more testing compared to males. Participants aged 18 or younger were less involved in testing during the pre-Delta period but increased their participation during the Delta period. Individuals aged 19-34 years underwent more testing in the pre-Delta period when compared to the Delta and post-Omicron periods. Participants who identified as Hispanics or Latinos had lower participation rates in post-Omicron (Table S1 in Multimedia Appendix 1 [29]).

### Demographic Characteristics of Test Participants

**Figure 3:**
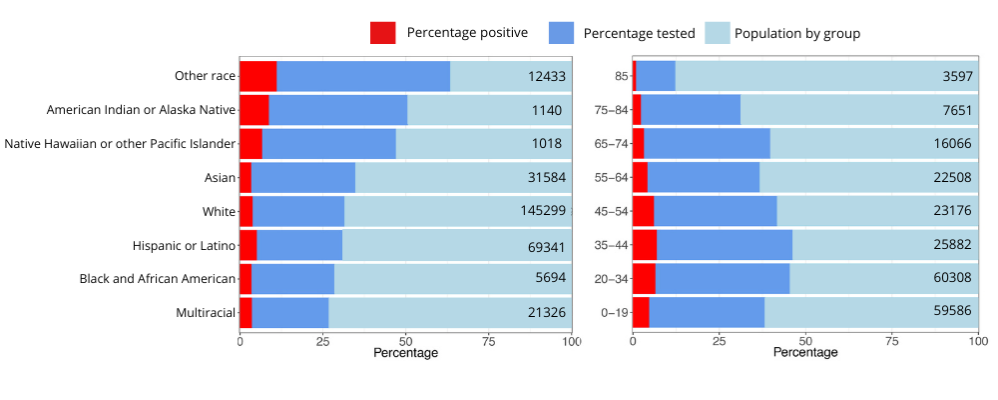
Rates of tests conducted and positive cases by race or ethnicity and age in years for Yolo County. The light-blue bars represent the total population size for each group based on the Census Bureau 2020 data. Dark-blue bars correspond to the proportion of participants within each demographic group. Red bars correspond to participants with a positive test result.

A total of 48,670 participants identified as female, making up 45.9% of the 106,034 estimated female population in Yolo County. Meanwhile, 39,524 were male, representing 35% of the 112,759 estimated male population in the county. Testing in all race or ethnic groups and age groups younger than 85 years exceeded 54,698 (25%) of Yolo County’s population (Figure 3). The lowest testing participation rate was observed among individuals aged older than 85 years. This is not unexpected, given that testing for participants aged 85 years and older was compulsory at congregate living facilities funded by Medicare or Medicaid, as part of several state-level testing initiatives. The highest overall test positivity rates were observed among individuals of American Indian or Alaska Native descent (7.6%), Native Hawaiian or Other Pacific Islander (5.6%), Latinos (4.5%), and people aged 20-44 years (20-34 years, 6.5%; 35-44 years, 6.9%).

### Adherence to Testing

**Figure 4:**
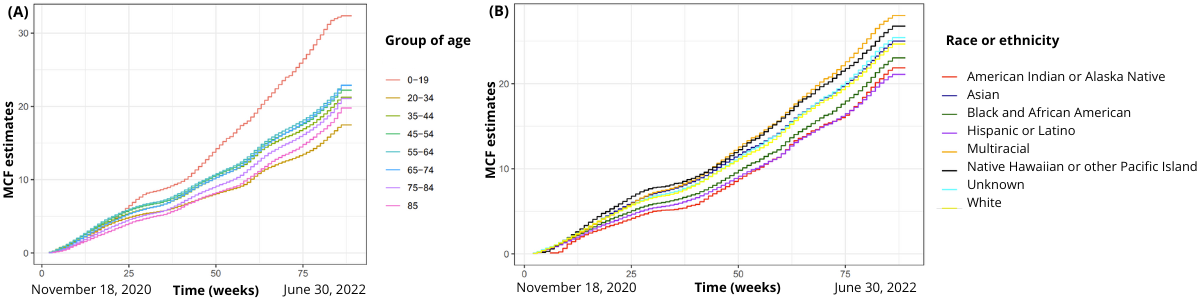
The MCF among different ages (A) and races (B). MCF represents the average accumulation of tests by age and race or ethnicity. MCF: mean cumulative functions.

In a sample of 61,363 participants that tested during this study period, there were 652,232 tests, with an average number of 10.62 (SD 13) tests per individual, in a median follow-up time of 40 (IQR 41.5) weeks. The average number of tests observed per week (MCF) by age and racial or ethnic groups is described in Figure 4A. Participants between the ages of 0 and 19 years demonstrated more consistent engagement in testing compared to other age groups, maintaining a higher level of involvement throughout this study’s period. Participants identifying as multiracial exhibited higher levels of engagement in testing compared to those identifying as Hispanic, Latino, American Indian, or Alaska Native (Figure 4B). Throughout this study period, the average testing patterns remained consistent across various age and racial or ethnic groups.

**Figure 5:**
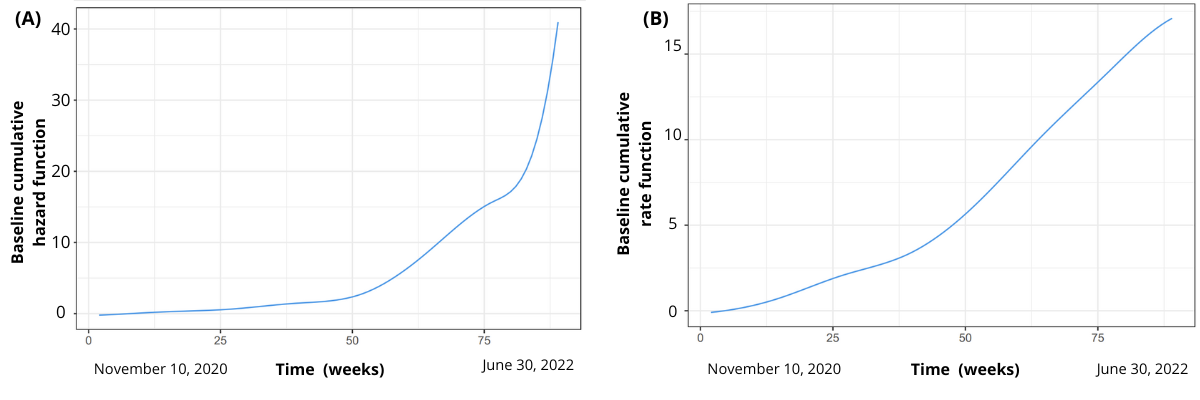
Baseline cumulative hazard function ((A) expected cumulative tests occurring up to a specific time) and baseline cumulative rate function ((B) expected tests up to a specific time).

Figure 5 illustrates the baseline cumulative rate function as the expected number of tests conducted up to a particular time point, independent of any covariates included in the model (Figure 5A). We also describe the baseline cumulative hazard function in recurrent tests, representing the expected cumulative number of tests occurring up to a specific time point, without considering any covariates or risk factors (Figure 5B). It provides a baseline estimate of testing occurrence over time.

### Adherence to Testing

The recurrent event analysis showed that male participants (HR 0.969, 95% CI 0.943-0.996) and individuals 85 years and older (HR 0.781, 95% CI 0.598-1.020) experienced lower adherence to testing compared to females and younger age groups (0-19 years), respectively (Table 2). Hispanic or Latino participants (HR 0.741, 95% CI 0.712-0.772) and American Indian or Alaska Native individuals (HR 0.735, 95% CI 0.567-0.953) experienced lower testing rates compared to individuals from other racial or ethnic groups.

**Table 2:** Covariate adjusted adherence to testing analysis.

Variable		Hazard ratio	95% CI	*P* value
**Sex (reference: female)**				
	Male	0.969	0.943-0.996	.002
**Age (years; reference: 0-19 years)**				
	20-34	0.861	0.828-0.895	<.001
	35-44	0.800	0.765-0.836	<.001
	45-54	0.840	0.806-0.876	<.001
	55-64	0.870	0.829-0.913	<.001
	65-74	0.782	0.750-0.816	<.001
	75-84	0.768	0.714-0.826	<.001
	85+	0.781	0.598-1.020	.01
**Race and ethnicity (reference: White)**				
	American Indian or Alaska Native	0.735	0.567-0.953	<.001
	Asian	0.912	0.873-0.953	<.001
	Black or African American	0.865	0.748-1.001	.008
	Hispanic or Latino	0.741	0.712-0.772	<.001
	Multiracial	0.988	0.928-1.052	.56
	Native Hawaiian or Other Pacific Islander	0.965	0.698-1.334	.70

### Discontinuation in Testing Participation

Male participants showed a significantly higher risk of discontinuing testing compared to females (HR 1.10, 95% CI 1.08-1.13), see Table 3. Individuals aged 20-34 years (HR 2.62, 95% CI 2.54-2.70) exhibited the highest hazard of discontinuing testing, followed by those aged 35-44 years (HR 1.51, 95% CI 1.47-1.56) and 45-54 years (HR 1.31, 95% CI 1.27-1.35), compared to those aged 0-19 years. Participants identifying as multiracial (HR 1.52, 95% CI 1.20-1.94), American Indian or Alaska Native (HR 1.20, 95% CI 1.05-1.36), and Hispanic or Latino (HR 1.19, 95% CI 1.15-1.22) were more prone to discontinuing testing compared to White participants. These findings underscore the importance of targeted outreach strategies tailored to engage and retain the demographic groups at risk of stopping testing. Addressing barriers such as accessibility, convenience, and perceived need for testing among younger age groups and specific racial or ethnic groups may help mitigate the discontinuation of testing and continue surveillance of SARS-CoV-2 transmission within communities.

**Table 3:** Covariate adjusted testing discontinuation analysis.

Variable			Hazard ratio		95% CI		*P* value
**Sex (reference: female)**							
	Male	1.104		1.075-1.134		<.001	
**Age (years; reference: 0-19 years)**							
	20-34	2.617		2.538-2.699		<.001	
	35-44	1.511		1.466-1.556		<.001	
	45-54	1.306		1.266-1.347		<.001	
	55-64	1.2		1.165-1.235		<.001	
	65-74	0.874		0.847-0.901		<.001	
	75-84	0.846		0.780-0.917		<.001	
	85+	1.185		0.936-1.501		.21	
**Race and ethnicity (reference: White)**							
	American Indian or Alaska Native	1.157		0.964-1.390		.27	
	Asian	0.932		0.889-0.976		.002	
	Black or African American	1.198		1.054-1.360		.02	
	Hispanic or Latino	1.185		1.148-1.223		<.001	
	Multiracial	1.523		1.198-1.935		.01	
	Native Hawaiian or Other Pacific Islander	0.969		0.908-1.034		.33	

## Discussion

HDT, and later HYT, was a community COVID-19 surveillance program that focused on free asymptomatic and symptomatic testing to catch cases early and provide resources and information for transmission reduction strategies. The primary focus of this study was to assess how well the program reached and maintained coverage and equity goals by tallying the demographic characteristics of participants and changes in testing participation. The HDT or HYT program used a high-throughput method to administer and process large volumes of tests, which allowed it to reach a substantial portion of the city of Davis and Yolo County’s population, covering all who willingly underwent testing. Extra testing campaigns and expanded access to free testing were adapted over time to ensure they reached the most vulnerable and underserved populations.

Although men typically face higher vulnerability to the health impacts of COVID-19 [30,31], the testing program saw a higher participation rate among women across all adult age groups except those aged 85 years or older. Additionally, men showed a slightly lower likelihood of retesting and a higher tendency to discontinue testing altogether compared to women. These results are consistent with research from prior infectious disease outbreaks indicating that women tend to be more cautious than men in the context of an epidemic [32,33] and more likely to adopt preventive behaviors and adhere to public health guidelines [34-36]. Additionally, studies have shown that women reported higher levels of fear regarding the coronavirus than men during the periods that HDT or HYT was active [7,37]. Gender differences in risk perception may stem from deeply ingrained gender roles or disparities in trust toward authority figures and institutions [2,38].

COVID-19 testing adherence varies across different age groups. Younger individuals (20-34 years) are less likely to retest and more likely to stop testing, while those aged 35-64 years have a higher probability of discontinuing testing. However, older age groups (65-74 years and 75-84 years) are less likely to stop testing compared to those aged 0-19 years. This pattern may be influenced by the free weekly testing provided to students aged 0-19 years by HDT or HYT in collaboration with multiple Yolo County school districts, and the mandatory testing for Medicare or Medicaid-funded congregate living facility residents supported by state-level testing programs.

The adherence to and cessation of testing also exhibit variations among various ethnic groups. Communities such as Hispanic or Latino and American Indian or Alaska Native tend to test less frequently compared to the White demographic. On the other hand, the Native Hawaiian or Other Pacific Islander group tends to test more frequently. There are also disparities in stopping testing, with Hispanic or Latino and Black or African American individuals more likely to cease testing compared to White individuals, while Asian individuals are less likely to do so. American Indian or Alaska Native and Multiracial groups show no significant difference. These variations could be due to differences in testing behaviors, access to testing facilities, or other community-specific factors. It is noteworthy that HDT or HYT has been conducting targeted outreach to heavily impacted communities and using data analysis to strategically allocate resources where increased testing is needed.

Monitoring the spread of a disease and accurately identifying its burden through a voluntary surveillance program relies heavily on maintaining consistent user cooperation and continuing recruitment of new users. However, disaster fatigue is a natural response during extended public health crises such as the COVID-19 pandemic that can reduce participation over time and stymy new recruitment efforts [15]. Nonetheless, HDT or HYT demonstrated the program’s effectiveness in retaining participants across a broad spectrum of age and race or ethnic demographics through and engaging new individuals in testing throughout its duration. The highest burden of infection arrived with the dominance of the Omicron variant in California, during which the testing program accelerated testing and outreach efforts to achieve the highest daily testing rate of the surveillance program. An overall increase in positive cases from Omicron is consistent with the drastic increase in virulence and avoidance of both immunity and contemporary prophylaxis [39-41]. Individuals who were already experienced with the system may have perceived increasing risk with the new variant, and opted for more testing; additionally, many workplaces and schools were requesting negative COVID-19 tests or proof of vaccination to return to the facility for a return to a sense of normalcy.

There was a significant decline in testing participation following the Omicron period. Motivation to engage in preventive behaviors against COVID-19 may have decreased because people became fatigued or burned out due to excessive and repeated exposure to similar messages about COVID-19 over time [15,16,21]. The increased availability, credibility, and convenience of other surveillance methods such as at-home COVID-19 tests [14], coupled with the impact of vaccines on risk perception [21], may have reduced the desire to continue with centralized surveillance testing. Lack of trust in government, misinformation, resistance, and conspiracy theories continue to pose a challenge for health authorities in maintaining or revitalizing public support for community-level surveillance of COVID-19, though it is unclear how this has affected participation in HDT or HYT.

In long-term crises such as the COVID-19 pandemic, public health authorities may constantly face new challenges in maintaining preventive strategies due to changes in the virus and social behavior. The comprehensive data collected during COVID-19 testing campaigns offers an opportunity to gain insight into people’s adherence and practices toward public health measures, which can inform the design of future strategies to educate communities about the benefits of engaging in preventive practices during new emerging infectious disease outbreaks.

The HDT or HYT program provides a unique setting to analyze people’s behavior since noncompliance with prevention measures may be due to reasons other than limitations in access or availability of tests. However, the nature of this data does not allow us to conclude the reasons for the observed differences. Nonetheless, the patterns observed in this analysis are consistent with findings reported in previous studies using different data sources, such as survey information.

The pandemic has highlighted the significance of social dynamics in disease control. Mathematical models played a crucial role in monitoring the behavior of SARS-CoV-2. However, access to information that accounts for social behavior and people’s adherence to health policies was extremely limited as was access to granular population-based incidence data. Analysis of acceptance and response is a starting point not only to improve future public health interventions but also to generate information that may be useful for the designing of statistical and mathematical models to study the dynamics of infectious diseases.

This analysis examines what factors affect acceptance to testing, using data from large-scale community PCR testing. While this type of data provides valuable insights, it falls short in capturing individual-level behavioral characteristics that may have affected the uptake of testing or vaccination resources. Collecting such data in future studies is crucial to optimizing the effectiveness of population-based interventions like testing, as it can help us identify the specific factors that influence people’s decisions to participate and inform the design of targeted interventions to improve uptake.

## Appendix

Multimedia Appendix 1 Demographic participation in testing.

## Abbreviations

HDT: Healthy Davis Together
HR: hazard ratio
HYT: Healthy Yolo Together
MCF: Mean Cumulative Function
PCR: polymerase chain reaction
