# Supplementary material for: Assessing SARS-CoV-2 Testing Adherence in a University Town: Recurrent Event Modeling Analysis
Source: JMIR Public Health Surveill. 2024 Apr 17;10:e48784. doi: 10.2196/48784 (PMC11025600; doi:10.2196/48784)
Supplement: Multimedia Appendix 1 [file publichealth_v10i1e48784_app1.pdf]

Supplement: Assessing SARS-CoV-2 Testing Adherence in a University Town: A Recurrent Event Modeling Analysis

Yury E. García, Alec J. Schmidt, Leslie Solis, Maria L. Daza–Torres,Cricelio Montesinos-López, Brad H. Pollock, Miriam Nuño

Table S1 summarizes the demographic characteristics of the participants by period and for the entire program, with direct comparison to the estimated population percentage in Yolo County from the ACS [29].

Table S1. Demographic Participation in Testing. The table summarize the percentage of participation for each demographic group among Participants, aggregated for the entire program and particular pandemic periods. The data is compared to the estimated population percentage in Yolo County from the American Community Survey (ACS) for contextual purposes.

Description	Yolo Pop N=218,774	Entire program N =89,924	Pre-Delta N=39,819	Delta N=58,920	Omicron N=46,545	Post-Omicron N=23,566	SD
**Sex**							
Female	51.5	54.1	54.6	54.9	54.9	55.0	0.2
Male	48.5	43.9	43.7	42.9	43.1	42.7	0.5
Unknown	-	1.9	1.6	2.2	2.1	2.3	0.3
**Age (years)**							
0-18	27.3	25.5	20.2	32.8	26.0	31.5	5.8
19-34	27.6	30.6	32.8	23.6	27.3	21.7	4.9
35-44	11.8	13.4	12.8	13.5	13.7	11.5	1.0
45-54	10.6	10.8	11.8	10.8	11.6	11.1	0.5
55-64	10.3	9.3	10.4	8.8	9.9	10.2	0.7
65-74	7.3	7.2	8.2	7.2	8.1	9.6	1.0
75-84	3.5	2.7	3.1	2.8	3.0	3.9	0.5
85	1.6	0.5	0.6	0.4	0.4	0.5	0.1
**Race**							
White	66.4	51.0	58.9	52.9	53.3	54.9	2.7
Asian	14.4	12.3	12.0	10.8	12.2	15.0	1.8
Black or African American	2.6	1.8	1.7	1.6	1.6	1.5	0.1
American Indian or Alaska Native	0.6	0.8	0.6	0.8	0.8	0.5	0.2
Native Hawaiian or Other Pacific Islander	0.5	0.5	0.4	0.5	0.5	0.3	0.1
Multiracial	9.7	6.4	6.8	6.7	6.6	6.9	0.1
Other Race	5.7	8.8	8.5	7.6	8.0	5.8	1.2
Unknown	-	18.4	11.1	19.1	17.0	15.1	3.4
**Ethnicity**							
Hispanic or Latino	31.7	23.9	20.7	22.7	21.9	16.2	2.9
Not Hispanic or Latino	68.3	58.5	70.6	58.6	61.4	68.4	5.7
Unknown	-	17.6	8.7	18.7	16.7	15.4	4.3

Yolo County Population (pop) baseline estimates were obtained from the 2020 American Community Survey.
SD: Standard deviation of the percentages in the four periods.

The participation rate is expressed as percentages by demographic group among the Participants in the entire program (N) and by period (N_i, i=1,2,3,4). Participation across different demographic groups was generally steady, with a few exceptions. Individuals under 18 years were underrepresented compared to their population during the Pre-Delta period (20.2% vs. 27.3% of the population), then sharply rose during the Delta period, after which they remained at or above their population percentage. Individuals between 19-34 years of age began in the Pre-Delta period with high testing contributions compared to their population (32.8% vs. 27.6% of the population), then dropped below their population percentage during the Delta and Post-Omicron periods (23.6% and 21.7%, respectively). Individuals self-identified as White showed an initial drop in participation after the Pre-Delta period (58.9% to 52.9% % from Pre-Delta to Delta) but held steady after that. Individuals who self-identified as Hispanic or Latino showed steady participation until the Post-Omicron period, where their participation showed a substantial drop (21.9% to 16.2% from Omicron to Post-Omicron). Individuals who did not volunteer race and ethnicity represent a large proportion of Participants (18.4% Overall), so comparisons with ACS estimates provide little additional information.
